# The ratio of propylene glycol to glycerol modulates nicotine taste and reward: more glycerol, more reward

**DOI:** 10.3389/fphar.2026.1955253

**Published:** 2026-09-23

**Authors:** Tyler Nelson, Alexxis Gutierrez, Karina Minanov, Merve Seferge, Nii A. Addy, Deniz Bagdas

**Affiliations:** 1 Department of Psychiatry, Yale School of Medicine, Yale University, New Haven, CT, United States; 2 Yale Center for the Study of Tobacco Product Use and Addiction, Yale University, New Haven, CT, United States; 3 Department of Cellular and Molecular Physiology, Yale School of Medicine, Yale University, New Haven, CT, United States

**Keywords:** e-cigarette, e-liquid, glycerin, palatability, propylene glycol, solvent, sweetness

## Abstract

**Background:**

Propylene glycol (PG) and glycerol (Gly) are odorless tobacco product constituents that act as solvents in electronic cigarette e-liquids and as humectants in oral nicotine products. Little is known about how PG/Gly ratio may affect nicotine’s sensory and rewarding properties. To address this gap, we utilized the taste reactivity test and four-bottle choice test to investigate the impact of PG/Gly ratio on nicotine’s taste and reward.

**Methods:**

A taste reactivity test was performed using young adult rats that received oral infusions of water, nicotine (10 μg/mL) and various PG/Gly ratios in the absence and presence of nicotine via intraoral catheters. Facial responses to each infusion were video recorded and the ingestive and aversive taste responses to each solution were scored and analyzed. In a separate cohort of rats, a four-bottle choice test was utilized to assess preference for various PG/Gly ratios with or without nicotine.

**Results:**

Young adult rats exhibited higher aversive reactions to PG100/Gly0 alone. Higher Gly content (PG0/Gly100 with nicotine) increased ingestive responses compared to nicotine alone. Higher Gly content (PG20/Gly80 with nicotine and PG0/Gly100 with nicotine) decreased aversive reactions compared to nicotine alone. Young adult rats also displayed greater preference for PG0/Gly100 alone and PG0/Gly100 with nicotine compared to vehicle water and nicotine, respectively.

**Conclusion:**

Overall, nicotine’s ingestive and aversive taste was altered by PG and Gly depending on the ratio. Specifically, higher Gly enhanced nicotine’s hedonic value and reduced nicotine’s aversive properties in both sexes. Higher Gly content was also preferred during the four-bottle choice test alone and in the presence of nicotine. Together, our results suggests that PG/Gly ratio, particularly high Gly content, influences nicotine’s abuse liability.

## 1 Introduction

Tobacco use remains a major public health issue worldwide. In recent years, alternative nicotine products such as electronic cigarettes (e-cigarettes) and oral nicotine products have rapidly gained popularity (Cornelius et al., 2023; Jamal et al., 2024). Alternative nicotine products differ from combustible cigarettes in both composition and delivery. Unlike traditional cigarettes, e-cigarettes produce aerosol by heating e-liquids (Talih et al., 2017; Yang et al., 2023) whereas oral nicotine products are chewed or absorbed through the oral cavity (Dennison Himmelfarb et al., 2025). These products typically contain nicotine and other constituents such as flavors, sweeteners, propylene glycol (PG) and glycerol (Gly) (Lee et al., 2020; Mallock-Ohnesorg et al., 2023; Sciuscio et al., 2022). While flavor additives have been shown to impact nicotine product appeal and use (Gades et al., 2022; Harrell et al., 2017; Leventhal et al., 2019), PG/Gly has been reported to impact nicotine delivery, flavor intensity, throat hit and vapor density (Harvanko et al., 2018; Li et al., 2016; Rao et al., 2018; Spindle et al., 2018). However, there are mixed results on the impact of PG/Gly on palatability, satisfaction and reinforcement value. It is not clear how PG vs. Gly (PG/Gly ratio) affects taste, the rewarding properties of nicotine, and nicotine use behaviors.

Sensory experience plays a critical role on nicotine product use, appeal, and satisfaction (DiPiazza et al., 2020; Goldenson et al., 2016; Peraza et al., 2026). In addition to flavors, PG and Gly may be important factors that strongly affect nicotine’s sensory properties. One study reported that participants rated “good taste” as the most important factor when using and purchasing e-liquids for e-cigarettes (Harvanko et al., 2018). Additionally, participants from the same study reported effects such as large clouds, good taste, and good smell to be most associated with Gly whereas effects like dry mouth, sore throat, headache and dizziness were more closely associated with PG, which suggests these solvents may influence the sensory and reinforcing effects of nicotine products (Harvanko et al., 2018). In another study, participants reported that the PG only e-liquid was significantly less “pleasant” and less “satisfying” compared to higher Gly e-liquids (Spindle et al., 2018). However, despite these reports on PG and Gly’s effects on the sensory attributes of e-cigarettes, one study found that neither PG or Gly was related to reinforcing effects (Harvanko et al., 2019). Furthermore, another study reported that PG/Gly ratio had minimal impact on any subjective effects or reinforcement value in ENDS naive current smokers (Smith et al., 2020b). Overall, existing research on PG/Gly ratio has focused largely on e-cigarette vapor, with inconsistent ratios across studies and no preclinical investigation. As a result, current findings on PG/Gly’s influence on palatability, satisfaction, and reinforcement are inconclusive and the specific effects of different ratios on the orosensory properties of nicotine and nicotine choice behavior has yet to be determined.

Here, we systematically examined whether different PG/Gly ratios alter taste responses to nicotine and whether the orosensory effects of PG/Gly influence choice behavior in rats. This study is the first to examine the effects of a wide range of PG/Gly ratios (0/0, 100/0, 80/20, 70/30, 50/50, 30/70, 20/80, 0/100) on the orosensory properties of nicotine, and the impact of low, medium, and high PG/Gly ratios (100/0, 50/50, 0/100) on nicotine choice behavior. We employed a taste reactivity test to assess the hedonic (ingestive) and aversive taste values and the four-bottle choice test to examine oral choice behavior in response to PG/Gly and nicotine. We hypothesized that higher Gly content compared to PG will enhance nicotine’s hedonic value and oral reward while decreasing aversion. Our findings showed that higher Gly content increased nicotine’s hedonic taste and decreased the aversive taste. Additionally, high Gly content increased oral nicotine reward (preference), providing evidence that Gly may play a critical role in influencing nicotine’s palatability and rewarding effects.

## 2 Methods

### 2.1 Animals

Young adult male (300–330g upon arrival) and female (250–275g upon arrival) Sprague-Dawley rats (Charles River Laboratories, Wilmington, MA, United States) were used. Rats were individually housed on a 12-h light/dark cycle in a room that was temperature and humidity controlled. Rats had free access to food (Envigo Bioproducts Inc rodent diet; Cat #NC1434463) and water throughout the study. The study was approved by the Yale University Institutional Animal Care and Use Committee (Protocol No. #2022-11366) and conducted according to the National Institutes of Health Guide for the Care and Use of Laboratory Animals.

### 2.2 Nicotine and PG/Gly preparation

Nicotine free base liquid was purchased from Sigma-Aldrich (Cat #N3876). PG was purchased from Sigma-Aldrich (Cat #W294004), and Gly was purchased from J.T Baker (Cat #02002926). Solutions were prepared using laboratory tap water, adjusted to a pH of 7.0, with NaOH and HCl used to stabilize the pH levels. The PG/Gly mixtures were prepared alone and in the presence of nicotine at the following ratios: 0/0, 100/0, 80/20, 70/30, 50/50, 30/70, 20/80, and 0/100. The 0/0 ratio consisted of water only and served as the vehicle control. Previous dilution experiments were performed to determine the preference for PG/Gly at different dilutions (10x – 100x; data not shown). Based on these experiments, our PG/Gly mixtures were first diluted 40 times in water, nicotine was subsequently added to these 40x-diluted PG/Gly solutions. All solutions were prepared fresh, wrapped in foil to protect them from light, stored at 4 °C and used within 3 weeks. On the test day, solutions were removed from the refrigerator and set out at room temperature 30 min prior to use in behavioral experiments. At the end of the day, any remaining solutions were discarded. We selected 10 μg/mL nicotine concentration, which we previously found to modulate nicotine’s taste and oral preference in rats (Bagdas et al., 2024; Bagdas et al., 2021). All concentrations are expressed as the free base form of nicotine.

### 2.3 Taste reactivity test

All rats were surgically implanted with a chronic oral catheter, to allow for solution delivery into the oral cavity during the test. Prior to surgery, rats were anesthetized with ketamine HCl (100 mg/kg, intraperitoneal) and xylazine (10 mg/kg, intraperitoneal). During the surgical procedure, we implanted the intraoral catheter laterally through the first maxillary molar, superficially between the ears. Carprofen analgesia (5 mg/kg, subcutaneous) was administered once prior to the surgery and then once daily for 3 days thereafter. Taste reactivity experiments began 5–7 days after recovery of the surgery.

On the day of the taste reactivity test, rats were placed in a rectangular plexiglass apparatus (30 × 12 × 22 cm). A mirror and digital video (Camcorder Sony HDR-CX240 and GoPro HERO8 Black, used at 30 FPS) camera were placed below the apparatus to record a bottom-up view of the rat’s orofacial responses. During the 45-min taste reactivity test, rats received 20 random 4-s infusions (200 μL of solution per infusion) of a test solution which was controlled by a computer program (Med Associates, Fairfax, VT). Rats were tested for only one solution during each session per day. Based on intraoral catheter patency, the taste reactivity protocol was limited to a maximum of eight test sessions, as extending testing beyond this period increases catheter failure and loss of animals from the study. All experiments were conducted during the light cycle every other day.

The test solutions were delivered intraorally via a surgically implanted catheter. Taste responses were evaluated, based on orofacial responses. Taste responses were categorized as hedonic (ingestive) or aversive. Tongue protrusions, lateral tongue movements, and rhythmic mouth movements were scored as ingestive responses. Gapes, headshakes, and forelimb flails were scored as aversive responses as described previously (Grill and Norgren, 1978). The present study examined the hedonic and aversive orosensory properties of PG/Gly ratio alone, with the addition of nicotine and the impact of different PG/Gly ratios on nicotine’s taste. Two separate cohorts of rats were used for the taste reactivity experiments. The first cohort was tested with water and seven PG/Gly ratios without nicotine, for a total of eight test solutions. The second cohort was tested with nicotine alone and seven nicotine-containing PG/Gly ratios, also for a total of eight test solutions. Within each cohort, a within-subjects design was used, such that each rat was tested with all eight solutions assigned to that cohort across eight sessions conducted every other day over approximately 15 days. The water control condition was tested in the first cohort, and these water control data were also used as the control comparison for analyses of the nicotine-containing solutions tested in the second cohort. Rats were counterbalanced across test solutions and received a randomized series of solutions. To examine potential sex differences in the taste reactivity of these solutions, both male and female rats were used in each cohort. Exclusion criteria and final group sizes are detailed below in Video Scoring and Statistical Analysis.

### 2.4 Four-bottle choice test

Rats were individually housed after their arrival, and they were habituated to their home cages for 5 days. To assess preferences for PG/Gly ratio, a four-bottle choice test was performed to provide the subjects with a choice between low, medium, and high PG/Gly content (PG0/Gly0, PG100/Gly0, PG50/Gly50, PG0/Gly100). Two water bottles were always present in the home cage, except during the four-bottle choice test. On test days, animals underwent a 4-h period of water deprivation, immediately prior to the four-bottle choice experiment, to induce a thirsty state. The rats were then tested in the custom-built four-bottle choice cages as described previously (Bagdas et al., 2026b). The testing cages were identical to their home cages, except that lids were built from stainless steel to allow for the placement of four 25 mL plastic tubes with rubber stoppers and stainless-steel drinking spouts.

During the final 10 min of the water deprivation period, the animals were transferred from their home cage to the empty testing cages, without bedding or food, for a 10-min habituation period. Following the habituation period, tubes containing test solutions were filled and placed in the testing cages in a randomized order, to prevent positional bias for each rat. The volume of each solution was measured in milliliters at the start of the experiment and again after 1 h, at the end of the experiment. The amount of each solution consumed by the animals was recorded as fluid intake (mL) per solution. Data were reported as the percent preference for each solution, which was calculated based on these fluid intake measurements. Preference for the test solution was determined by dividing the volume of test solution consumed by the total volume consumed and multiplying by 100. To account for any spillage that might naturally occur, an empty cage was used every test session, equipped with four bottles of test solutions. If any spillage occurred in the empty cage, the corresponding amount of spillage was removed from the total volume in the test cage during analysis. A between-subjects design was used for these experiments.

### 2.5 Video scoring and Statistical Analysis

Videos from the taste reactivity test were labeled and coded by lab personnel following the completion of experiments. An experimenter (TN, AG, KM, MS) blind to the experimental condition scored the 6 s following each infusion for each solution. Data were collected by taking the summation of ingestive or aversive responses for each scored infusion. These summed responses were then averaged across the total infusions for each animal and plotted as the number of ingestive or aversive responses for each of the experimental conditions.

Animals were excluded from analysis if the animal’s mouth was out of view for more than 2 of the 6 s in the post-infusion scoring window. Additionally, animals with blocked or obstructed oral catheters were excluded from further testing. As a result of these exclusion criteria, final group sizes varied by condition and cohort. The taste reactivity cohorts initially included 8 rats per sex in the nicotine-free groups and 10 rats per sex in the nicotine-containing groups. For the PG/Gly alone cohort, 5 to 6 rats per sex were included in the final data set. For the PG/Gly with nicotine cohort, 6 to 9 rats per sex were included. The four-bottle choice test cohorts included 9 rats per sex in the nicotine-free condition and 11 rats per sex in the nicotine-containing condition. All animals were included in the final data set.

All data were analyzed using Graph Pad Prism (Version 10; GraphPad Software, Inc., San Diego, CA). Assumptions of normality were tested with the Shapiro-Wilk test prior to performing an ANOVA. Taste reactivity data (n = 5–9) were analyzed using a mixed-effects repeated measures analysis of variance (RM ANOVA). When appropriate, a Greenhouse-Geisser correction was applied (α = 0.05). Significant main effects were further examined using Dunnett’s *post hoc* tests, and planned comparisons between water and nicotine alone were analyzed using unpaired t-tests. An additional mixed-effects two-way ANOVA was used to assess potential sex differences across PG/Gly ratios in the absence and presence of nicotine. Šídák’s multiple comparisons test was used for *post hoc* comparisons between males and females within each test solution. Four-bottle choice data (n = 9–11) were analyzed using a two-way ANOVA. Significant main effects were further analyzed using Bonferroni-corrected multiple comparisons. Statistical significance was determined at *p* < 0.05.

## 3 Results

### 3.1 PG/Gly ratio alone does not alter ingestive taste responses

To first determine the hedonic (ingestive) and aversive taste responses to PG/Gly alone, we investigated the taste responses to different PG/Gly ratios without nicotine and compared with water. Intraoral infusions of water and different PG/Gly ratios elicited ingestive taste reactivity responses (Figure 1). In females, there was no main effect of ratio for ingestive responses (*F*_(2.284, 11.750)_ = 0.654, *p* = 0.557, Figure 1A). Similarly, there was no main effect of ratio on ingestive responses in male rats (*F*_(1.570, 6.954)_ = 1.694, *p* = 0.247, Figure 1B), and there was no main effect when data from male and female rats were combined (*F*_(3.441, 33.920)_ = 1.301, *p* = 0.290, Figure 1C). Overall, there were no differences in ingestive taste responses among the different PG/Gly ratios versus water in females, males, or when female and males were combined. Additional analysis of potential sex differences revealed no significant main effect of sex (*F*_(1, 72)_ = 2.489, *p* = 0.1191), no significant main effect of group (*F*_(3.250, 33.42)_ = 1.300, *p* = 0.2909), and no significant sex and group interaction) *F*_(3.250, 33.42)_ = 0.5546, *p* = 0.6621) for ingestive responses.

**FIGURE 1:**
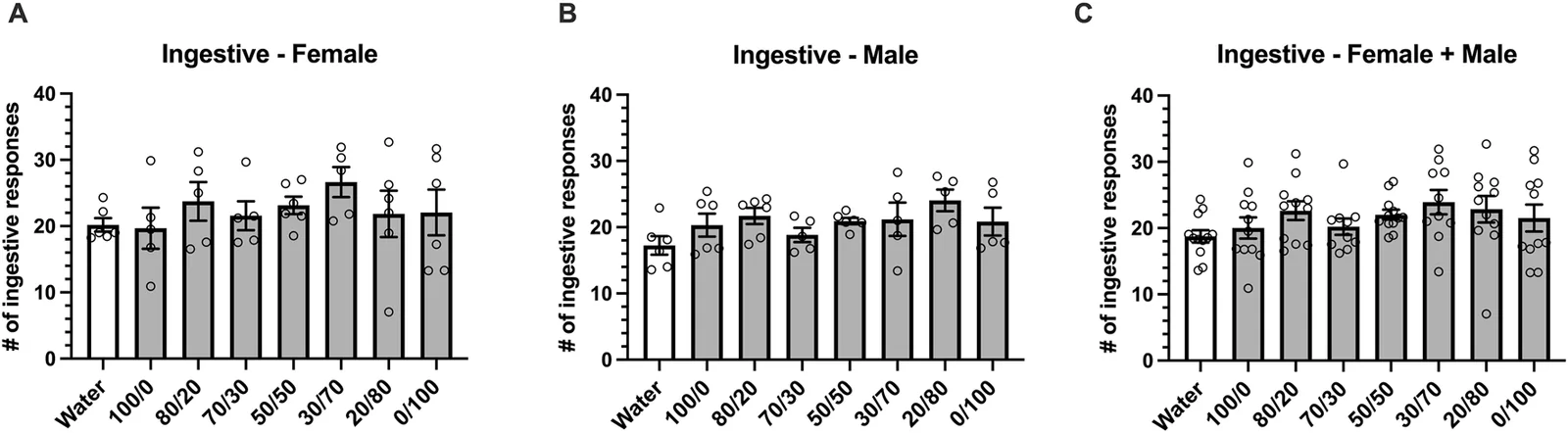
Effects of PG/Gly ratio on hedonic taste reactivity responses. **(A)** Ingestive taste responses to PG/Gly ratio in females, n = 5–6 rats per condition. **(B)** Ingestive taste responses to PG/Gly ratio in males, n = 5–6 rats per condition. **(C)** Ingestive taste responses to PG/Gly ratio in female and male rats combined, n = 10–12 rats per condition. Data are expressed as mean ± standard error of the mean (SEM).

### 3.2 High propylene glycol content (PG100) alone increases aversive taste responses

Intraoral infusions of water and varying PG/Gly ratios also elicited aversive taste reactivity responses (Figure 2). In females, there was a significant main effect of ratio on aversive responses (*F*_(1.933, 8.561)_ = 4.530, *p* = 0.0464, Figure 2A). Although Dunnet’s *post hoc* test showed no significant effect of individual ratios, a trend towards increased aversive responses to the highest PG content (PG100/Gly0) was found, while the other ratios were similar to the water control. In male rats, there was no main effect of ratio on aversive responses (*F*_(1.120, 4.959)_ = 3.942, *p* = 0.103, Figure 2B). When data from both sexes were combined, a mixed-effects one-way ANOVA revealed a significant main effect of ratio on aversive taste reactivity responses (*F*_(2.396, 23.61)_ = 6.324, *p* = 0.004 Figure 2C). Dunnett’s *post hoc* test revealed a significant increase in aversive responses to PG100/Gly0 compared with water (*p* = 0.039), while the other ratios did not alter aversive responses compared with the water control. Additional analysis of potential sex differences revealed no significant main effect of sex (*F*_(1, 10)_ = 0.0486, *p* = 0.830), significant main effect of group (*F*_(1.870, 16.56)_ = 7.116, *p* = 0.0066), and no significant sex and group interaction (*F*_(1.870, 16.56)_ = 1.436, *p* = 0.2645) for aversive responses.

**FIGURE 2:**
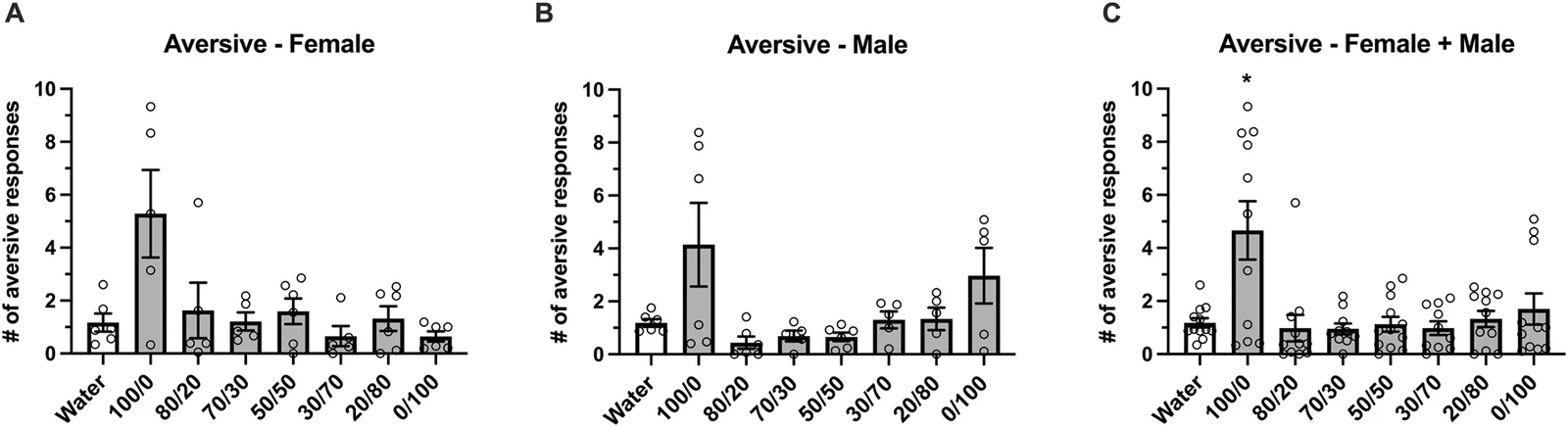
Effects of PG/Gly ratio on aversive taste reactivity responses. **(A)** Aversive taste responses to PG/Gly ratio in females, n = 5–6 rats per condition. **(B)** Aversive taste responses to PG/Gly ratio in males, n = 5–6 rats per condition. **(C)** Aversive taste responses to PG/Gly ratio in female and male rats combined, n = 10–12 rats per condition. Data are expressed as mean ± SEM. *p < 0.05 versus water.

### 3.3 High glycerol content (Gly100) increases nicotine’s ingestive taste responses

To determine the impact of PG/Gly ratio on nicotine’s ingestive taste, a wide range of PG/Gly ratios were examined in the presence of nicotine during the taste reactivity test (Figure 3). A water control was also examined to investigate the ingestive and aversive taste responses to nicotine alone. In females, a planned comparison between water and nicotine, revealed that nicotine elicited fewer ingestive responses compared with water (*p* = 0.019). Additionally, a mixed-effects one-way RM ANOVA revealed a significant main effect of ratio for ingestive responses (*F*_(3.292, 31.46)_ = 2.962, *p* = 0.036, Figure 3A). Female rats exhibited higher ingestive responses to PG0/Gly100 with nicotine compared to nicotine alone (*p* = 0.011).

**FIGURE 3:**
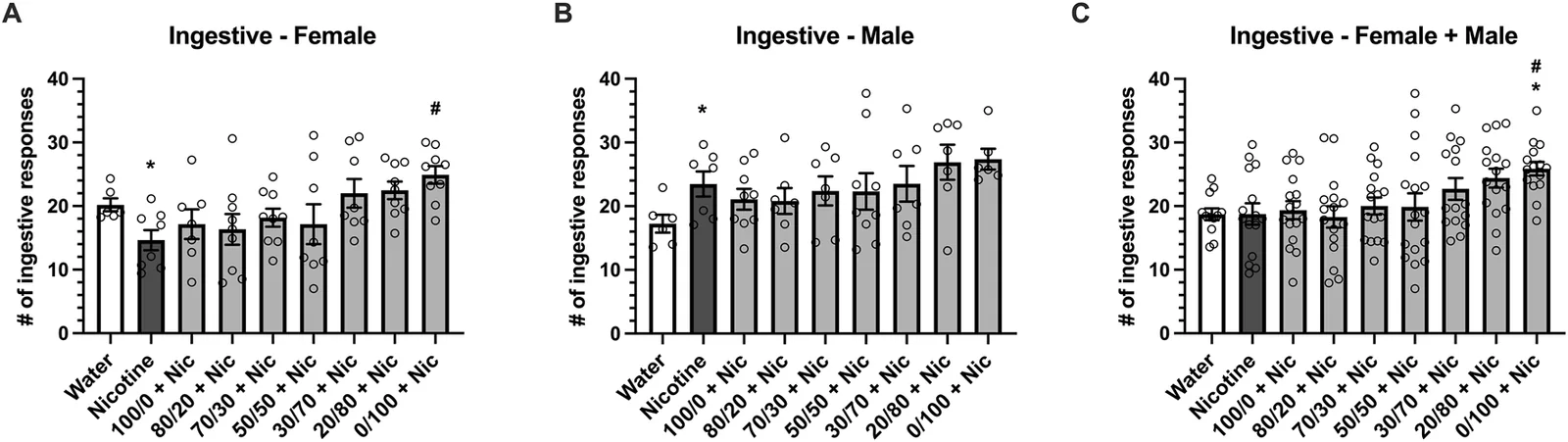
Effects of PG/Gly ratio on nicotine’s hedonic taste reactivity responses. **(A)** Ingestive taste responses to water, nicotine and PG/Gly ratios in the presence of nicotine in female rats, n = 6–9 rats per condition. **(B)** Ingestive taste responses to water, nicotine and PG/Gly ratios in the presence of nicotine in male rats, n = 6–9 rats per condition. **(C)** Ingestive taste responses to water, nicotine and PG/Gly ratios in the presence of nicotine in female and male rats combined, n = 12–17 rats per condition. Data are expressed as mean ± SEM. *p < 0.05 versus water. #p < 0.05 versus nicotine.

In males, a planned comparison between water and nicotine, revealed that nicotine elicited higher ingestive responses compared with water (*p* = 0.029). A mixed-effects one-way RM ANOVA revealed that there was no main effect of ratio on ingestive responses (*F*_(3.590, 21.54)_ = 2.063, *p* = 0.127, Figure 3B). In contrast to females, males showed no differences in the ingestive responses to the different PG/Gly ratios with nicotine; however, there was a trend toward increased ingestive responses with higher Gly content.

Additional analysis of potential sex differences revealed significant main effect of sex (*F*_(1, 16)_ = 6.437, *p* = 0.0220), significant main effect of group (*F*_(4.669, 60.69)_ = 3.719, *p* = 0.0063), and no significant sex and group interaction (*F*_(4.669, 60.69)_ = 1.126, *p* = 0.3552) for ingestive responses. Post hoc tests revealed significantly greater ingestive responses in males than females for nicotine alone (*p* = 0.0379), with no significant sex differences for the other test solutions. When both sexes were combined, a mixed-effects one-way RM ANOVA revealed a significant main effect of ratio for ingestive responses (F _(5.347, 75.520)_ = 3.767, *p* < 0.004, Figure 3C). Post hoc tests revealed significant increases in the ingestive responses to PG0/Gly100 compared with water (*p* = 0.014) and nicotine alone (*p* = 0.010).

### 3.4 High glycerol content (PG20/Gly80 and PG0/Gly100) decreases nicotine’s aversive taste responses

To determine the impact of PG/Gly ratio on nicotine’s aversive responses, PG/Gly ratios were examined in the presence of nicotine during the taste reactivity test (Figure 4). A water control was also tested to determine aversive responses to nicotine, in the absence of PG/Gly, and to allow for additional comparisons. In females, a mixed-effects one-way RM ANOVA revealed a significant main effect of ratio on aversive responses (*F*_(3.715, 26.01)_ = 7.174, *p* < 0.001, Figure 4A). Post hoc tests revealed that female rats exhibited significantly more aversive responses to nicotine alone (nicotine in PG0/Gly0) and to PG/Gly ratios 100/0, 80/20, 70/30, 50/50, and 30/70 with nicotine, compared with water (*p* < 0.05). Additionally, PG/Gly vehicle at ratios of 20/80 and 0/100 combined with nicotine elicited significantly lower numbers of aversive responses compared to nicotine alone (*p* < 0.05).

**FIGURE 4:**
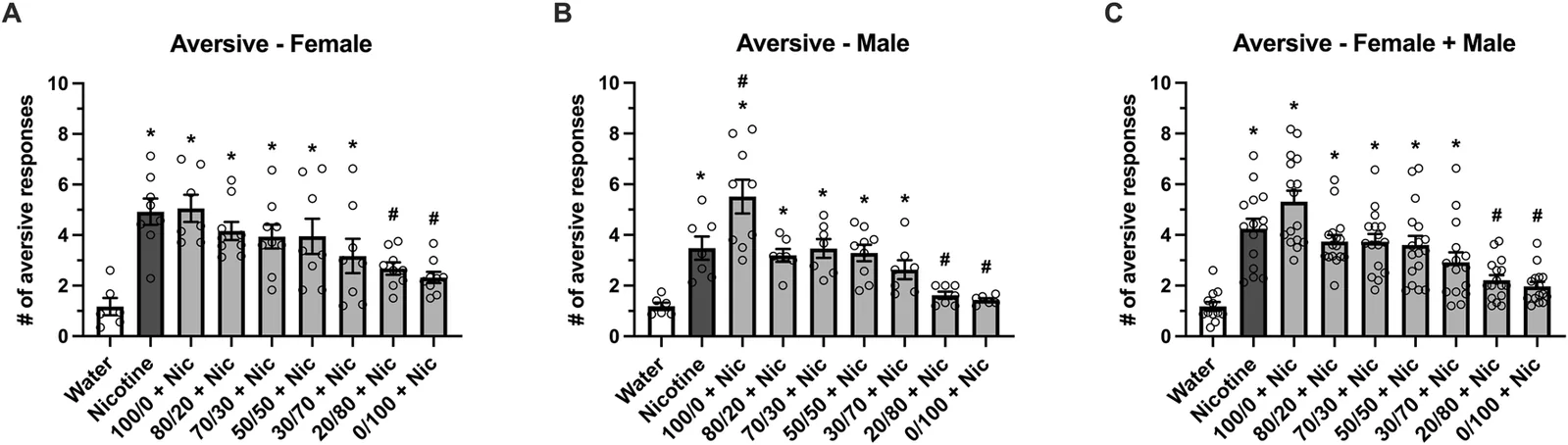
Effects of PG/Gly ratio on nicotine’s aversive taste reactivity responses. **(A)** Aversive taste responses to water, nicotine and PG/Gly ratios in the presence of nicotine in female rats, n = 6–9 rats per condition. **(B)** Aversive taste responses to water, nicotine and PG/Gly ratios in the presence of nicotine in male rats, n = 6–9 rats per condition. **(C)** Aversive taste responses to water, nicotine and PG/Gly ratios in the presence of nicotine in female and male rats combined, n = 12–17 rats per condition. Data are expressed as mean ± SEM. *p < 0.05 versus water. #p < 0.05 versus nicotine.

In males, a mixed effects one-way RM ANOVA revealed a significant main effect of ratio on aversive responses (*F*_(3.143, 18.86)_ = 15.22, *p* < 0.0001, Figure 4B). Post hoc tests revealed that male rats exhibited a significant increase in aversive responses to nicotine alone and to nicotine combined with PG/Gly at ratios 100/0, 80/20, 70/30, 50/50, and 30/70, compared with water (*p* < 0.05). Male rats also exhibited increased aversive responses to nicotine combined with PG100/Gly0 compared with nicotine alone (in PG0/Gly0), whereas nicotine combined with PG/Gly ratios of 20/80 and 0/100 elicited fewer aversive responses compared with nicotine alone (*p* < 0.05).

Additional analysis of potential sex differences revealed no significant main effect of sex (*F*_(1, 16)_ = 5.327, *p* = 0.0347), significant main effect of group (*F*_(4.457, 57.94)_ = 18.11, *p* < 0.0001), and no significant sex and group interaction (*F*_(4.457, 57.94)_ = 1.064, *p* = 0.3860) for aversive responses. Post-hoc test revealed significantly greater aversive responses in females than males for nicotine combined with PG20/Gly80 (*p* = 0.0234) and nicotine combined with PG0/Gly100 (*p* = 0.0329) groups, with no significant sex differences for the other test solutions.

When both sexes were combined, a mixed-effects one-way RM ANOVA revealed a significant main effect of ratio on aversive responses, (*F*_(4.872, 68.820)_ = 15.07, *p* < 0.0001, Figure 4C). Post hoc tests revealed higher aversive responses to nicotine alone and higher aversive response to PG/Gly ratios of 100/0, 80/20 70/30, 50/50, and 30/70 combined with nicotine, compared with water (*p* < 0.05). When female and male rat data was combined, data analysis revealed that adult rats also exhibited significantly fewer aversive responses to PG/Gly ratios 20/80 and 0/100 combined with nicotine, compared with nicotine alone (in PG0/Gly0; *p* < 0.05).

### 3.5 High glycerol content (Gly100) increases reward in nicotine choice behavior

To determine the effects of PG/Gly ratio on nicotine choice behavior, we selected a low, medium, and high ratio (0/0, 100/0, 50/50, and 0/100) to examine effects on preference, either with PG/Gly alone and with PG/Gly combined with nicotine (Figure 5). The percent preference to various PG/Gly ratios without nicotine is shown in male and female rats (Figure 5A). A two-way ANOVA revealed a significant main effect of ratio (*F*_(3, 64)_ = 4.914, *p* = 0.0039) and a no main effect of sex (*F*_(1, 16)_ = 0.00, *p* = 0.9995; Figure 5A). Post hoc tests revealed a significant preference for 0/100 compared to water (0/0) in male rats (*p* < 0.05), whereas female rats showed no difference in preference between PG/Gly (0/100) compared to water (0/0).

**FIGURE 5:**
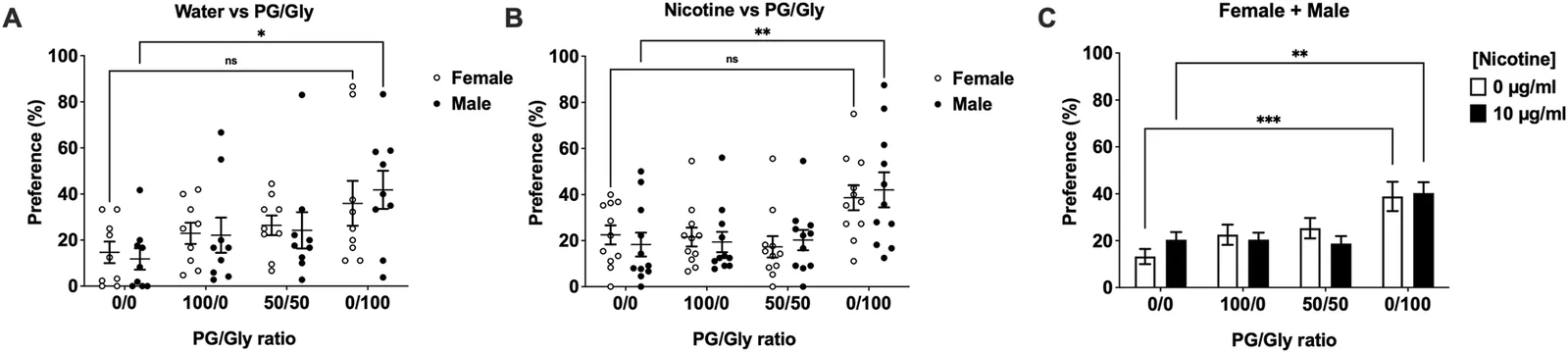
Effects of PG/Gly ratio in isolation and in the presence of nicotine on preference (reward) in the four-bottle choice test. **(A)** Percent preference for water versus PG/Gly ratio in female and male rats, n = 9 per condition. **(B)** Percent preference for nicotine versus PG/Gly ratio in the presence of nicotine in female and male rats, n = 11 per condition. **(C)** Percent preference for water versus PG/Gly ratio in isolation and in the presence of nicotine in female and male rats combined. Data are expressed as mean ± SEM. *p < 0.05, **p < 0.01, ***p < 0.001.

Figure 5B shows the percent preference for various PG/Gly ratios containing nicotine in male and female rats. A two-way ANOVA revealed a significant main effect of ratio (*F*_(3,80)_ = 8.078, *p* < 0.0001, Figure 5B) and no main effect of sex (*F*_(1,80)_ = 0.00, *p* > 0.9999). Post hoc tests revealed a significant preference for the highest Gly content (0/100) compared with nicotine (0/0) in male rats (*p* < 0.05), but female rats showed no significant difference. When data from female and male rats were combined, a two-way ANOVA revealed a significant main effect of ratio (*F*_(3, 152)_ = 12.16, *p* < 0.0001, Figure 5C), but no main effect of nicotine (*F*_(1, 152)_ = 0.00, *p* = 0.9996) on preference. Post hoc tests revealed that both PG0/Gly100 alone and PG0/Gly100 combined with nicotine resulted in significantly greater preference in adult rats compared with water (0 μg/mL) and nicotine (10 μg/mL), respectively (p < 0.05).

## 4 Discussion

In this study, we investigated the effects of PG/Gly ratio on nicotine’s taste and reward across a wide range of ratios. PG/Gly alone showed similar ingestive taste responses with water, however, increasing the PG content induced aversive taste responses compared with water. In the presence of nicotine, increasing the Gly content enhanced the ingestive but decreased the aversive taste responses to nicotine, while increasing PG content intensified nicotine’s aversive taste responses. In addition to taste, higher Gly content increased nicotine reward in our choice behavior paradigm. Together, these findings provide the first systematic examination of PG/Gly ratio effects, in isolation and in combination with nicotine, on nicotine’s taste and reward in rodents.

Non-nicotine e-cigarette use is common among youth, especially during initiation. Among lifetime e-cigarette users, who never smoked cigarettes, 55.6% initiated with nicotine-free e-cigarettes and among past-year adolescent vapers, 66% reported using e-cigarettes without nicotine (Krishnan-Sarin et al., 2015; Tokle et al., 2021). This raises the question of what drives e-cigarette use in the absence of nicotine. One possibility is the sensory experience itself, which users commonly report as a motivator for using e-cigarettes (DiPiazza et al., 2020; Goldenson et al., 2016; Peraza et al., 2026; Rao et al., 2018). PG/Gly, the solvent base in e-liquids, may influence not only the sensory experience but the motivation to use e-cigarettes, with or without nicotine. Indeed, recent work from our lab found that PG/Gly vapor alone resulted in robust vapor self-administration behavior in rats, suggesting that the solvent vehicle may have its own reinforcing effects (Graham et al., 2026). Together, these findings highlight the importance of characterizing the sensory and rewarding properties of PG/Gly in isolation and in combination with nicotine to better understand the non-nicotine factors that may impact nicotine product use.

A critical and understudied variable is the ratio of PG to Gly in e-liquids and nicotine products. Commercial e-liquids are available across a wide range of PG/Gly ratios, with common formulations including PG30/Gly70, PG50/Gly50 and PG20/Gly80 (Ma et al., 2023). In their sample of 14,407 e-liquid products, PG30/Gly70 was the most prevalent (50%) followed by PG50/Gly50 (14%), PG20/Gly80 (5%) and PG25/Gly75 (4%), other (8%) and missing (19%). Despite the ubiquity of PG/Gly in nicotine products, the taste responses elicited by different PG/Gly ratios have not been characterized.

Our results suggest PG/Gly ratio alone does not influence hedonic taste responding as demonstrated by the absence of any significant increases in ingestive responses across our PG/Gly ratios, but it does significantly influence aversive taste responses. This indicates that the solvent base itself is not sufficient to drive palatability, though it is possible that the sweetness and hedonic value of PG/Gly may emerge when combined with nicotine or when heated into vapor. Our aversive finding that PG alone (PG100) can elicit aversive orosensory responses is consistent with previous literature linking high-PG vapor to aversive effects such as dry mouth, sore throat, headache, dizziness, and harshness (Harvanko et al., 2018; Spindle et al., 2018), suggesting our taste reactivity findings translate to the subjective effects reported by human users. Our findings provide preclinical evidence that PG/Gly ratio, independent of nicotine, can alter the orosensory experience, specifically with regards to aversive taste.

Next, our findings that nicotine alone produced sex-specific effects on ingestive responses and heightened aversive responses in both sexes relative to water, are broadly consistent with prior work from our laboratory examining nicotine’s taste effects across a range of concentrations (1–100 μg/mL, Bagdas et al., 2021). Nicotine (10 μg/mL) reduced hedonic responding in our female rats which aligns with our prior study that similarly found reduced ingestive responses to nicotine (10 μg/mL in females, 30 μg/mL in males). However, our aversive findings that nicotine (10 μg/mL) significantly increases aversive responses in both sexes diverge from our earlier work where aversive responses emerged only at higher concentrations in males (10 μg/mL in females, 30 μg/mL in males). This discrepancy may be explained through a methodological difference between the two studies. Animals from our prior work (Bagdas et al., 2021) were exposed to a wide range of nicotine concentrations conditions testing as high as 100 μg/mL, which could independently alter aversive taste responding. In contrast, the present study held nicotine at a constant concentration of 10 μg/mL across all conditions, varying only the PG/Gly ratio.

PG/Gly ratio significantly influences both ingestive and aversive taste responses to nicotine in a ratio-dependent manner, with the highest Gly content ratio (PG0/Gly100) increasing the palatability of nicotine, an effect that was most pronounced in female rats. The observed sex effect likely reflects the hedonic value of nicotine alone in each sex where males showed increased ingestive responses to nicotine compared with water, leaving less room for Gly to further enhance hedonic responding. In contrast, females showed suppressed ingestive responses to nicotine alone which increasing Gly content (PG0/Gly100) appeared to restore in females.

Our data in the current study indicate that PG/Gly ratio systematically shifts the taste profile of nicotine, with higher Gly enhancing ingestive responses and attenuating aversion while higher PG content exacerbates nicotine’s aversive taste. Specifically, the highest PG content ratio (PG100/Gly0) worsened nicotine’s aversive taste effects in males, while the highest Gly content ratios (PG20/Gly80 and PG0/Gly100) reduced aversive responses to nicotine in both sexes. Our preclinical findings align with and extend the existing human literature on PG/Gly effects: high Gly content has been linked to greater palatability, pleasantness, and positive sensory attributes such as good taste and smell (Harvanko et al., 2018; Spindle et al., 2018), whereas high PG content is associated with aversive sensory effects such as dry mouth, sore throat, and dizziness (Harvanko et al., 2018). Consistent with this, PG-only e-liquids were rated as significantly less pleasant and satisfying than Gly-only formulations (Spindle et al., 2018).

Furthermore, the ability of high Gly content to increase hedonic responding and reduce nicotine aversion effects is similar to what has been previously observed with sweeteners such as sucrose (Grill and Norgren, 1978; Wilmouth and Spear, 2009) raising the possibility that Gly may be acting through its sweetness value. In prior taste reactivity test work, sweet-associated compounds increase ingestive responses and show no or less aversion, while bitter-associated compounds are associated with heightened aversive responses (Grill and Norgren, 1978; Wilmouth and Spear, 2009). Our Gly taste reactivity findings may be explained through considering Gly a sweet-associated compound. Electrophysiological recordings in the gerbil demonstrate that Gly directly stimulates gustatory taste receptors, consistent with its classification as a sweet tastant (Jakinovich and Goldstein, 1976). A study in humans examining the perceived sweetness of various solutions established that Glycerol’s perceived sweetness is approximately half that of sucrose (Moskowitz et al., 1974). There have also been reports in previous literature and online forums describing Gly as having a slightly sweet taste relative to PG (Rao et al., 2018). While direct scientific characterization of Gly’s sweet taste in the context of inhalation or intraoral delivery remains limited, the convergence of gustatory receptor evidence, human sweet perception data, and user-reported sensory properties suggests that Gly’s sweet taste is a plausible mechanism underlying our observed taste reactivity test effects. Our taste reactivity findings demonstrate a shift toward increasing hedonic responding and attenuating aversion with increasing Gly content, which is consistent with the addition of a palatable, sweet-tasting substance. Prior work from our laboratory further demonstrates that flavor additives can modulate nicotine’s orosensory profile in the test reactivity test paradigm. Menthol, for example, increased ingestive and decreased aversive responses to nicotine in male rats, and benzaldehyde attenuated nicotine’s aversive responses in females at 10 μg/mL nicotine, the same concentration used in the current study (Bagdas et al., 2021). Together, these previous findings establish that the orosensory experience of nicotine and flavors supporting the plausibility that Gly may also be enhancing the palatability of nicotine-containing solutions similar to sweeteners and flavors.

It is important to note, however, that the present study was not designed to directly test this sweetness hypothesis, and the mechanistic basis of Gly’s modulatory effects on nicotine taste remains to be established. Other properties of Gly, including its viscosity, humectant properties, and potential effects on vapor characteristics, may also contribute to the observed palatability-enhancing effects and cannot be excluded. Future studies employing sweet taste receptor knockout models or pharmacological blockade of sweet taste signaling will be necessary to determine whether Gly’s effects on nicotine’s orosensory profile are mediated through sweet taste pathways. The present findings raise the possibility that higher Gly content may enhance nicotine palatability through a possible sweetness-associated mechanism, with important implications for nicotine abuse liability.

Beyond PG/Gly effects on taste, PG/Gly ratio influenced nicotine reward in the four-bottle choice test paradigm, with the highest Gly content ratio (PG0/Gly100) producing the greatest increase in preference, both alone and combined with nicotine. This finding directly parallels our taste reactivity results, in which the highest Gly content ratio produced the most pronounced increase in ingestive responses, strengthening the interpretation that PG/Gly ratio may be influencing nicotine’s hedonic and rewarding properties through a shared taste mechanism. These results are in line with the broader hypothesis that sweet tastants enhance nicotine reward, as we recently reported that saccharin similarly increases nicotine reward (preference) in the four-bottle choice test paradigm (Bagdas et al., 2026b). In a recent study for our lab, we also found that PG55/Gly45 has its own reinforcing effects in our vapor self-administration paradigm (Bagdas et al., 2026a). This is consistent with other preclinical reports of PG/Gly reinforcement. In a similar model, rats self-administering vehicle (PG50/Gly50) vapor alone showed lever discrimination, responding more on the active versus the inactive lever, suggesting that the PG50/Gly50 mixture used was reinforcing on its own (Smith et al., 2020a). Our taste reactivity and nicotine choice behavior results may offer a potential mechanism for these self-administration findings, as Gly’s ability to reduce nicotine aversion and enhance ingestive responding may underlie the reinforcing effects of PG/Gly vapor observed across these studies. In contrast, human laboratory findings have reported minimal impact of PG/Gly on reinforcement value (Harvanko et al., 2019; Smith et al., 2020b), though variations in route of exposure, device characteristics, and methodology may explain this difference.

In the absence of nicotine, PG/Gly ratios did not produce significant sex differences in either ingestive or aversive responses of taste reactivity test. In contrast, sex differences emerged when nicotine was present. Females showed significantly greater aversive responses than males to nicotine combined with PG20/Gly80 and PG0/Gly100, whereas males showed significantly greater ingestive responses than females to nicotine alone. In the four-bottle choice experiments, no significant sex differences were observed in preference for the different PG/Gly ratios, either in the absence or presence of nicotine. These findings suggest that sex differences may be limited to orosensory responses to nicotine-containing solutions in taste reactivity tests.

In summary, the present study is the first to report the effects of PG/Gly ratio on nicotine’s taste and rewarding properties. While PG/Gly alone at all ratios had similar hedonic value compared with water, PG100/Gly0 elicited aversion. Increasing the Gly content (PG0/Gly100) enhanced the hedonic taste value of nicotine, reduced its aversive taste value, and potentiated oral nicotine reward. On the other hand, the highest PG content (PG100/Gly0) ratio exacerbated the aversive taste of nicotine. Our convergent findings across nicotine’s taste and reward suggest that Gly content can enhance nicotine palatability. Given that solvent composition can shape nicotine’s sensory and rewarding properties, these findings carry direct implications for tobacco product regulation. Tobacco regulatory authorities should consider PG/Gly ratio in product standards and labeling, with particular attention to Gly content as a contributor to enhancing nicotine’s abuse liability. Future studies should examine the specific mechanism underlying Gly’s effects, including the potential role of sweet taste signaling, and determine whether these findings extend to inhaled nicotine delivery and human participants.

## Data Availability

The original contributions presented in the study are included in the article/supplementary material, further inquiries can be directed to the corresponding author.
